# Development of Passive Fire Protection Materials Based on Calcium Magnesium Phosphate Cements and Perlite

**DOI:** 10.3390/ma19010069

**Published:** 2025-12-24

**Authors:** Georgiana-Florina Badea, Alina-Ioana Badanoiu, Georgeta Voicu, Roxana Trusca, Adrian-Ionut Nicoara

**Affiliations:** 1 Department of Science and Engineering of Oxide Materials and Nanomaterials, Faculty of Chemical Engineering and Biotechnologies, National University of Science and Technology Politehnica Bucharest, 1-7 Gh. Polizu, 011061 Bucharest, Romania; 2 National Research Center for Micro and Nanomaterials, National University of Science and Technology Politehnica Bucharest, 313 Spl. Independenţei, 060042 Bucharest, Romania

**Keywords:** calcium magnesium phosphate cement, dolomite, passive fire protection materials, perlite

## Abstract

Calcium magnesium phosphate cements (CMPCs) were obtained starting from dolomite (alone or mixed with fly ash) thermally treated at two different temperatures. Dolomite calcination at 750 °C for 3 h determined the formation of a mixture of MgO and CaCO_3_. The mixing of dolomite with fly ash and the increase in the calcination temperature at 1200 °C determined the formation of new compounds (calcium aluminum silicate and calcium magnesium silicates), which are present along with MgO and small amounts of CaO in the thermally treated material. These two precursors were mixed with KH_2_PO_4_ solution and borax (as a retardant admixture) to obtain the CMPCs. The setting time and compressive strengths of these CMPCs were assessed and the XRD analyses provided insights into their mineralogical composition after hardening and thermal treatment. The cements, as so or mixed with perlite, were applied on steel plates, to assess their behavior when put in direct contact with a flame. The compatibility of these materials with the steel substrate was evaluated by scanning electron microscopy (SEM). The direct contact with the flame up to 60 min provided information regarding the CMPCs’ ability to prevent the rapid increase in the substrate (steel plate) temperature. The findings indicate that CMPC pastes and composites containing perlite can offer a degree of protection for steel structures in the event of a fire.

## 1. Introduction

Magnesium phosphate cements (MPCs) have attracted increasing attention due to their rapid setting, high early strength, and good fire-resistant properties. These cements are particularly promising for fire protection due to their thermal stability, good adhesion to substrates, and eco-friendly characteristics [1,2,3].

Recent studies have highlighted the superior fire resistance of MPCs. MPCs exhibit excellent performance under high temperatures and can maintain structural integrity, preventing steel substrates from reaching critical failure temperatures (~500–600 °C) during fire exposure [4,5]. The fire resistance is attributed to the formation of stable crystalline phases like struvite or K-struvite (KMgPO_4_·6H_2_O), which dehydrate endothermically and absorb heat. Additives such as fly ash, glass powder, or glass fibers can enhance MPCs’ fire resistance [6,7].

Calcined dolomite is increasingly being studied as a substitute for magnesia in magnesium phosphate cements, offering both cost-effective and environmental benefits due to the lower temperature at which the thermal treatment is performed (750–1300 °C) as compared with magnesite (over 1500 °C) [8,9,10]. Calcium magnesium phosphate cements (CMPCs) can be synthesized from calcined dolomite and phosphate solutions through a process involving acid–base chemistry. Depending on the thermal treatment temperature, dolomite (CaMg(CO_3_)_2_) decomposes into a mixture of MgO and CaCO_3_ if the temperature is 600–800 °C [9] or MgO and CaO if the temperature is above 1000 °C [10]. These oxides act as the basic components in the cement formulation when mixed with phosphoric acid or an aqueous solution of phosphate salts (such as ammonium dihydrogen phosphate or potassium dihydrogen phosphate).

Due to the high reactivity of calcium oxide (CaO) and its highly exothermic reaction with phosphate solutions, the direct use of CaO in calcium magnesium phosphate cements (CMPCs) can lead to undesirable effects such as rapid setting and volume instability [10]. When using calcined dolomite as a dead burned magnesia substitute, it is essential to control the reactivity of the resulting CaO. One effective strategy is to introduce minerals containing oxides, such as silica (SiO_2_) and/or alumina (Al_2_O_3_), during the calcination process [10,11]. These oxides can chemically react with CaO to form stable, less reactive compounds, thereby reducing its solubility and moderating the heat release during cement formation. This approach enhances the compatibility of calcined dolomite with phosphate-based systems and improves the overall performance of the resulting cement.

In a previous study, we have evaluated the efficacy of CMPCs based on dolomite thermally treated at 750 °C to protect the steel substrate during fire exposure [9].

The coating formulated with calcined dolomite demonstrated satisfactory adhesion to steel substrate under normal conditions. However, during fire testing involving direct flame exposure, significant coating delamination from the metal surface was observed. While these initial findings are promising, they highlight the need for further investigation. One potential development is to explore a range of additives to enhance the performance of calcium magnesium phosphate cements (CMPCs), with a focus on fire behavior.

Enhancing the porosity of paste, mortar, or concrete improves their ability to provide thermal insulation. One method used to increase the porosity of magnesium phosphate cements is the use of foaming agents. Lai et al. [12] obtained MPCs with high porosity (63.2% to 74%) using a chemical foaming agent (based on sodium dodecyl sulfate, alkyl amido betaine, and citric acid) and Zn powder. The amount of Zn powder had the highest influence on the MPCs’ porosity. Xie et al. [13] obtained foamed MPCs with low thermal conductivity (0.109 to 0.098 W·m^−1^·K^−1^) using calcium stearate to generate porosity; sodium bicarbonate [14] and H_2_O_2_ [15] were also employed as foaming agents to enhance the porosity of the hardened MPC.

A second approach to increasing MPC porosity is to incorporate lightweight materials into the MPC formulation, such as expanded vermiculite [5,16], porous alkali-activated material based on waste glass [6], or expanded perlite [6,17]. The incorporation of these porous materials in MPCs improved fire resistance but resulted in a reduction in compressive strength. According to Fang et al. [17], impregnating expanded perlite with a silane solution could help mitigate the reduction in the compressive strength of MPCs.

Building on the initial findings [6,9], this study aimed to further develop calcium magnesium phosphate cements (CMPCs) using dolomite calcined under two different conditions: at 750 °C and at 1200 °C. In the latter case, to mitigate the high reactivity of calcium oxide (CaO) formed during calcination, dolomite was blended with fly ash prior to thermal treatment. This new approach was intended to promote the formation of less reactive calcium-containing phases. Additionally, to enhance the thermal insulation properties of the resulting CMPCs, expanded perlite was incorporated into selected formulations. These modifications were designed to improve not only the thermal performance but also the overall stability and applicability of the coatings under high-temperature conditions.

This study represents an original approach to design and experimentally validates this specific type of formulation aimed at developing fire-retardant coatings for steel structures.

## 2. Materials and Methods

**Figure 1:**
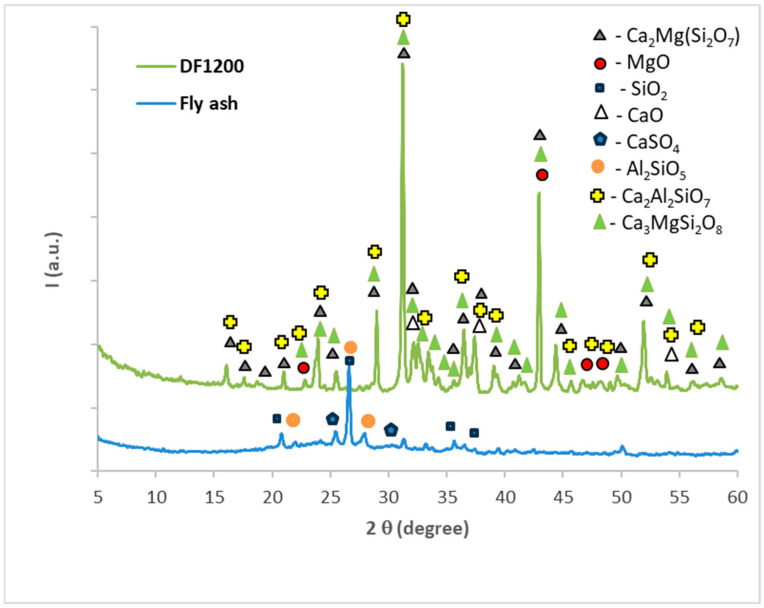
XRD patterns of fly ash and DF1200 (dolomite + fly ash thermally treated at 1200 °C, for 3 h).

The following materials were used in this study:

- Commercially available, dead burned magnesia (M) from Tremag, Tulcea, Romania, industrially obtained by the thermal treatment of magnesite (MgCO_3_) at 1500 °C. The oxide composition of dead burned magnesia comprises 94.05% MgO; 2.52% SiO_2_; 1.98% CaO; 0.68% Fe_2_O_3_; 0.4% Al_2_O_3;_ and loss on ignition is 0.37%.
- Fly ash, waste generated by coal burning in a thermal power plant (Romania). The main crystalline compounds assessed by XRD were quartz (SiO_2_), Al_2_SiO_5,_ and CaSO_4_—Figure 1. The mean particle size is 4.8 microns [4].
- Natural dolomite (D) (Rodbungrup, Bucharest, Romania) containing 47% CaCO_3_ and 37.5% MgCO_3_. XRF analysis also showed the presence of impurities including Si = 1.3%; Al = 0.728%; Fe = 0.551%; and other minor elements (0.35%).

This dolomite (D) was thermally treated, in a Nabertherm electrically heated chamber furnace (Lilienthal, Germany), in two different conditions:

‑ D750—Thermal treatment at 750 °C for 3 h; this thermal treatment determines the decomposition of CaMg(CO_3_)_2_ in MgO and CaCO_3_ [9];
‑ Dolomite (D) was mixed with fly ash (F) and the mixture was thermally treated at 1200 °C for 3 h. This material was denominated DF1200.

The mixing of the dolomite and fly ash (weight ratio 2:1) and the thermal treatment at 1200 °C for 3 h leads to the decomposition of CaMg(CO_3_)_2_ with the formation of CaO and MgO and the partial reaction of these oxides with the compounds present in the fly ash; this leads to the formation of a calcium aluminum silicate—Ca_2_Al_2_SiO_7_ (PDF 04-016-0209)—and calcium and magnesium silicates, i.e.—Ca_3_MgSi_2_O_8_ (PDF 01-086-3096)—and Ca_2_MgSi_2_O_7_ (PDF 04-015-7956)—Figure 1.

**Figure 2:**
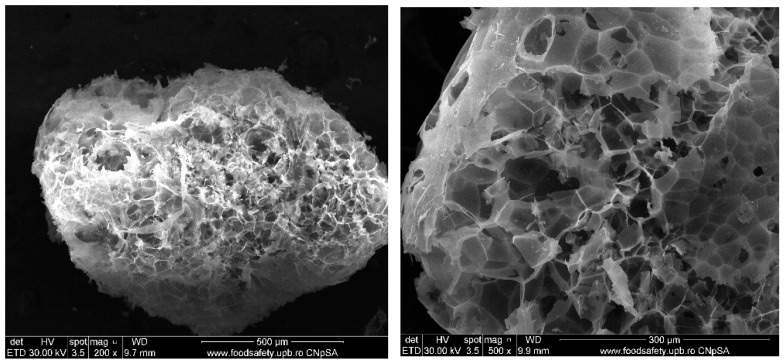
SEM images of expanded perlite (EP).

- Commercially available (Procema perlit, Jilava, Romania), expanded perlite—(EP) with particle sizes below 2 mm, density (dry in loose state) comprised between 40 and 65 kg/m^3^, and low thermal conductivity (max. 0.042 W·m^−1^·K^−1^)—according to the product data sheet. This material contains round particles with high porosity (Figure 2) resulting from the thermal treatment of natural perlite, due to the water release [6,18].
- KH_2_PO_4_ (P) and Na_2_B_4_O_7_∙10H_2_O (B)—Chemical reagents from Sigma-Aldrich (Darmstadt, Germany).

Table 1 presents the compositions of MPC and CMPCs.

To improve the mechanical properties and setting time of the D750-based phosphate cement, in this study, a lower D750/phosphate ratio (1.5) was used, compared to the one used in our previous research [9].

The setting time of the cement pastes was determined using a Vicat apparatus equipped with a Vicat needle—Matest, Treviolo, Italy (SR EN 196-3 [19]). Two setting times were determined as follows:

- The initial setting time refers to the period between mixing the cement components and the moment when the Vicat needle stops at 1 mm measured from the base plate.
- Final setting time—the time interval from the mixing of the components until the needle forms a slight indentation on the surface of the cement paste.

The phosphate cement pastes (Table 1) were cast into cylindrical molds (Φ = 16 mm and h = 16 mm) cured for 24 h and then demolded. Following this, the specimens were kept in air at ambient temperature. The compressive strength of these specimens was determined after 3, 7, 28, and 610 days of air curing using a Matest Cyber-Tronic testing machine (Matest, Treviolo, Italy); the average value of the compressive strength was calculated based on at least three values, assessed on specimens cured under similar conditions.

The paste specimens cured for 7 days were subjected to a thermal treatment simulating fire condition [20], characterized by a rapid temperature rise to 1200 °C (15 min), followed by a plateau of 30 min, and cooling with a 20 °C/min rate. To perform this thermal treatment, the specimens (cylinders) were vertically placed in an electrically heated furnace with a controller (Nabertherm, Lilienthal, Germany).

Following the thermal treatment, compressive strength was determined using a Matest Cyber-Tronic testing machine (Matest, Treviolo, Italy). The loading rate was 0.5 MPa/s. Also, the thermally treated specimens were finely ground, and the resulting powder was analyzed by X-ray diffraction.

The compositions M_B_P, D750_B_P, DF1200_P, and D750_3EP_B_P were cast onto steel plates (length = 100 mm; width = 100 mm; height = 2.7 mm) for fire testing purposes.

Sandpaper was used to grind the steel plate surface before applying the coatings.

These compositions were applied in layers: an initial layer of M_B_P followed by a second layer of either D750_B_P, DF1200_P, or D750_3EP_B_P. Additionally, the D750_3EP_B_P composition was also cast directly onto the steel plate. To achieve coatings with larger thickness (10–11 mm), a polystyrene mold was employed during casting and the initial hardening phase (first day), after which it was removed. The samples were allowed to cure for 7 days, after which they were exposed to direct flame contact to monitor the temperature of the metallic substrate.

For the preliminary evaluation of fire behavior, the coated metal plates were exposed to a fire torch with butane flame, which was applied directly to the surface of the hardened cement paste. The plates were positioned vertically on a support, with the flame in direct contact with the coated face. The distance between the burner and plate was kept constant (3 cm) and all the tests were performed in the same ambient conditions. A detailed description of the testing method can be found in reference [21].

The temperature of the steel plate was recorded at 1-min intervals over 30 or 60 min on the opposite side of the cement coating, using an infrared thermometer with an accuracy of ±1% relative to the recorded temperature displayed on the digital screen of the device. An uncoated steel plate was used as a reference.

X-ray fluorescence (XRF) analysis was conducted using a Thermo Scientific ARL PERFORM’X Sequential XRF Spectrometer (Thermo Fisher Scientific, Waltham, MA, USA) on pressed powder specimens. The samples were prepared as 30 mm diameter discs via uniaxial compaction. Measurements were performed under vacuum conditions at room temperature. Quantification followed the UniQuant analytical protocol, and accuracy was assessed through comparison with certified reference standards.

To effectuate the X-ray diffraction (XRD) analysis, a Shimadzu XRD 6000 diffractometer (Shimadzu, Kyoto, Japan) with CuKα radiation (λ = 1.5406 Å) at a scanning rate of 2°/min was used. XRD patterns were recorded over a 2θ range of 10–60 degrees. The database used for the identification of XRD peaks was PDF-5+ 2025 from ICCD.

The microstructure and elemental composition of the cement pastes applied on the steel plates were analyzed using a QUANTA INSPECT F50 scanning electron microscope (FEI Company, Eindhoven, The Netherlands), equipped with a field emission gun (FEG) electron source, offering a resolution of 1.2 nm, and an energy-dispersive X-ray spectroscopy system with a resolution of 133 eV at the MnK line.

The coatings were applied to steel plates, cured in air for 7 days, and subsequently cut using a circular metal-cutting saw. SEM and EDX analyses were performed on freshly cut samples. To enable proper electrical conductivity during microstructural investigation, the samples were gold-coated for 60 s.

## 3. Results and Discussion

The setting times of the studied phosphate cements are presented in Table 2.

The data indicates that the magnesia-based phosphate cement (M_B_P) has a fast setting time even though a setting retarding admixture, i.e., borax (B) was used. According to the literature information, the retarding action of borax is due to the formation of an insoluble hydrate layer on MgO particles, thus hindering Mg^2+^ release and slowing the hydration process [22,23]. The borax dosage along with other parameters (such as Mg/P ratio, water to binder ratio, or ambient temperature) can also significantly influence the setting time of magnesium phosphate cements [3,23].

For the M_B_P composition, the rapid setting may be attributed to the low borax content, which does not significantly hinder the rapid formation of K-struvite (KMgPO_4_·6H_2_O) [6].

When the solid component is dolomite calcined at 750 °C, in the absence of the borax, the phosphate cement (D750_P) has a very short setting time; therefore, the borax was added to the cement composition (D750_B_P), and the initial setting time increased from 4 min to 53 min, and the final setting time from 7 min to 62 min. This important increase in the setting time could be an advantage for the use of these types of cement, as it provides sufficient time for its preparation and application.

**Figure 3:**
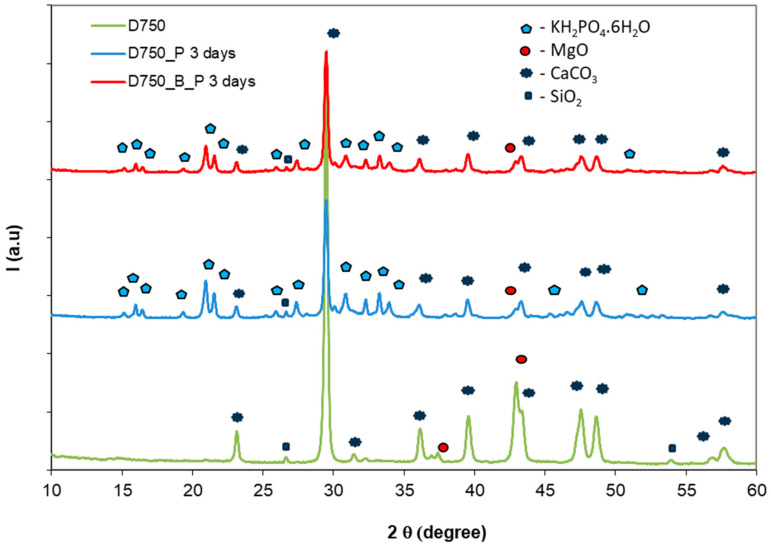
XRD patterns of phosphate cements based on thermally treated dolomite, without borax (D750_P) and with borax (D750_B_P), after 3 days of curing.

The mixing of thermally treated dolomite (D750) with the phosphate solution also leads to the formation of K-struvite. On the XRD patterns, shown in Figure 3, the peaks of K-struvite (PDF 01-090-9099) and calcium carbonate (PDF 01-086-4272) from D750 can be observed on the cement pastes hardened for 3 days.

The use of borax as a setting retarder does not modify the nature of hydrates formed in these compositions but only affects the rate of the setting process (see D750_P compared with D750_B_P).

When the solid component was DF1200, the use of borax in the preparation of the corresponding phosphate cement (DF1200_B_P) led to a significant setting delay. In the absence of borax (DF1200_P), this cement had an initial setting time of 70 min and a final setting time of 149 min.

**Figure 4:**
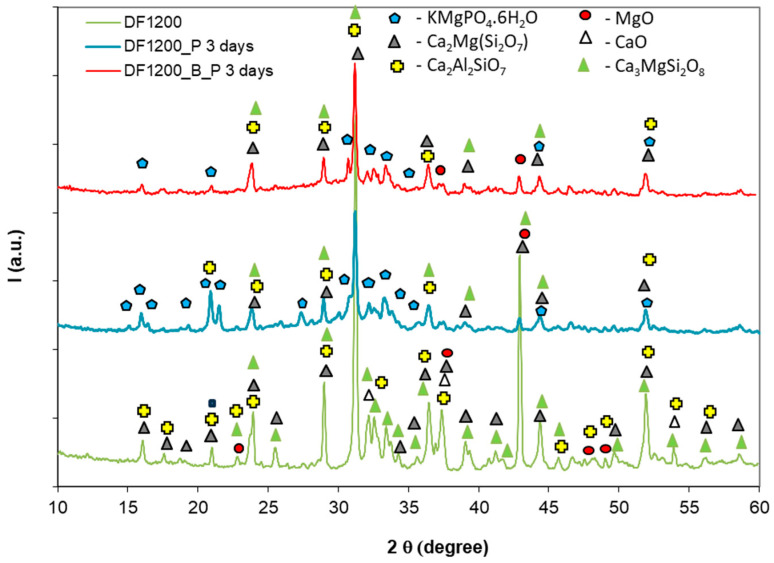
XRD patterns of the dolomite and fly ash mixture thermally treated at 1200 °C (DF1200) and corresponding phosphate cements without borax (DF1200_P) and with borax (DF1200_B_P) after 3 days of curing.

When DF1200 was used as a solid component for the preparation of the phosphate cement, the main compound assessed by X-ray diffraction was also K-struvite (KMgPO_4_·6H_2_O)—Figure 4. This phase is identified alongside the compounds already present in DF1200, i.e., gehlenite, merwinite, and akermanite. MgO present in the DF1200 composition is consumed in K-struvite formation. In this case as well, the use of borax does not modify the mineralogical composition of the sample, but only the kinetics of the setting process.

**Figure 5:**
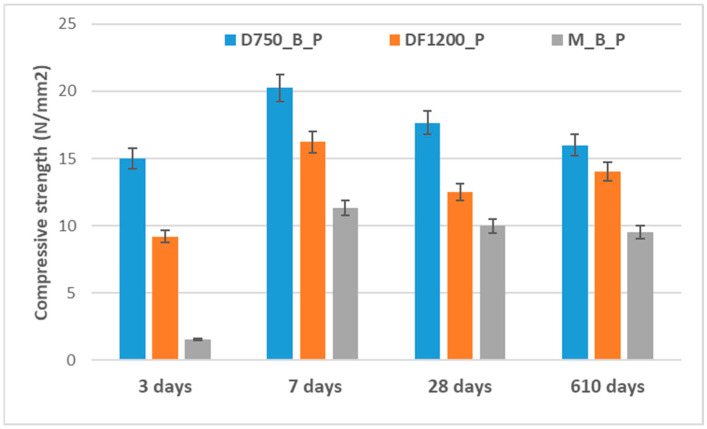
The time-dependent evolution of the compressive strength of studied cements cured in air.

The time-dependent evolution of the compressive strength of the studied phosphate cements, cured in air, is presented in Figure 5.

The compressive strengths developed by the investigated phosphate cements depend on the nature of the solid precursors, the reaction products formed, and the rate of processes leading to the hardening of these materials. The phosphate cement based on dolomite thermally treated at 750 °C (D750_B_P) develops compressive strengths higher than the one based on DF1200_P. Correlating the data with the values of the setting times (Table 2) and the XRD analyses presented in Figure 3 and Figure 4, it can be concluded that the formation of the K-struvite and the rate of this process are defining for the development of the compressive strength. Slower rates of interaction between the solid component and the phosphate solution, mediated by the presence of the borax, do not allow the development of an adequate quantity of K-struvite that would assure the growth of the compressive strengths at short periods of time; on the other hand, a much faster reaction between the two components (for example, the M_B_P mixture) could determine the occurrence of internal tensions that cause the decrease in compressive strength values as compared with D750_B and DF1200_B_P, even though the K-struvite quantity formed in the first case could be more significant. Although for the composition DF1200_P a small drop (23%) in compressive strength is recorded after 28 days of hydration (possibly due to the delayed hydration of traces of CaO still present this composition); no major changes in the compressive strengths are recorded after a much longer hardening time (610 days).

**Figure 6:**
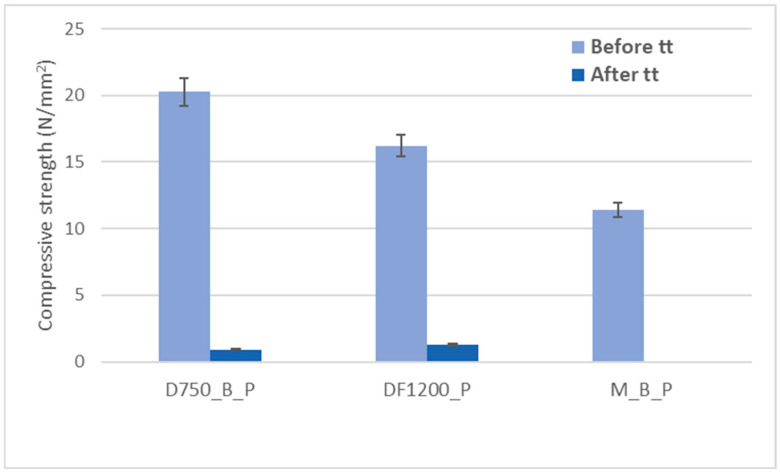
The influence of thermal treatment on the compressive strength of phosphate cements.

The effects of thermal treatment performed in the furnace on the compressive strengths of the investigated phosphate cements are shown in Figure 6.

As shown in Figure 6, the thermal treatment applied to the hardened cement pastes leads to a notable reduction in compressive strength, which could be partially due to the thermal shocks suffered by these specimens due to the rapid growth in the temperature. For the D750_B_P specimen, a decrease in the compressive strength of 95% was recorded, and for the DF1200 specimen, the decrease is 92.12%. Following thermal treatment, the M_B_P specimen exhibited no measurable compressive strength. The occurrence of cracks in the surface layer of the specimen M_B_P after the thermal treatment (Table 3) suggests that material segregation appears to have occurred due to the use of a high water to solid ratio (0.3). Therefore, further experiments were conducted with a reduced water content, using water to solid ratio of 0.2.

**Figure 7:**
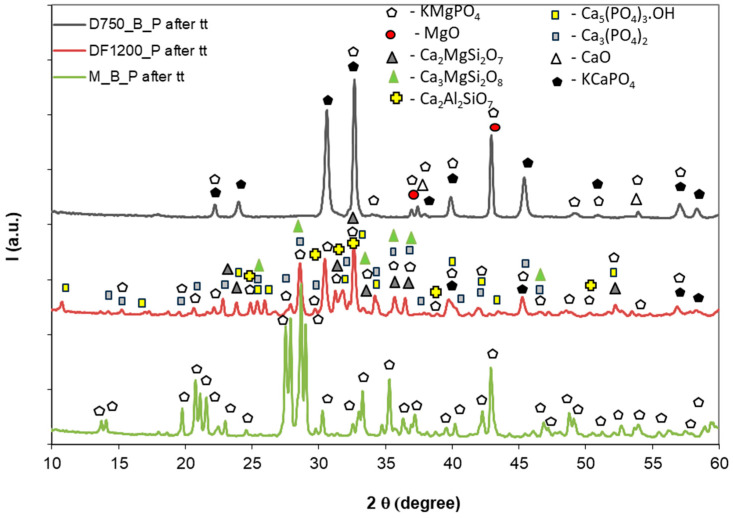
XRD patterns of thermally treated (tt) phosphate cements.

To assess the effect of thermal treatment on the mineralogical composition of the studied materials, XRD analysis was performed. The diffraction patterns are shown in Figure 7.

For the thermally treated M_B_P cement, one can notice the total dehydration of K-struvite with the formation of KMgPO_4_ (PDF 04-009-7824) [6]. For D750_B_P, the compounds identified were KMgPO_4_, KCaPO_4_ (PDF 01-091-8238), and MgO (PDF 00-004-0829). On the XRD patterns of the DF1200_P composition, after thermal treatment, one can identify the presence of gehlenite, merwinite, akermanite, KMgPO_4,_ and KCaPO_4_. On the XRD pattern of this specimen, one can also notice the presence of hydroxyapatite Ca_5_(PO_4_)_3_OH (PDF 04-010-6312) compound, which can result from the reaction of bioactive compounds (merwinite and akermanite) with phosphate solution [24,25,26]. Initially, it exhibits a low degree of crystallinity, but its crystallinity increases with the rising temperature. Since in this study the thermal treatment was performed at 1200 °C, hydroxyapatite begins to decompose, forming Ca_3_(PO_4_)_2_, as also confirmed by XRD [27,28]. Due to the rapid heating and short holding time (30 min) at 1200 °C, Ca_5_(PO_4_)_3_OH may still be present within the interior of the tested specimens (cylinders).

It can be concluded that, along with the previously mentioned thermal shock suffered by these specimens, due to the rapid increase in temperature, the dehydration of K-struvite is a key factor contributing to the observed strength loss [29]. The release of water vapors results in an increased number of pores and internal defects within the specimen [30,31]. The presence of other compounds, such as gehlenite, merwinite, and akermanite, helps to slightly mitigate this negative effect.

To assess the ability of these coatings in limiting the temperature rise in the steel substrate, the coated metal plates were exposed directly to flame. Previously obtained results revealed that phosphate cements based on D750 have poor adhesion to the metallic substrate after the fire test [9]; based on this information, in this study, the steel plate was first coated with a magnesium phosphate cement (M_B_P), which has a good adhesion to the metallic substrate [4], followed by the coating with a layer of phosphate cement based on thermally treated dolomite.

**Figure 8:**
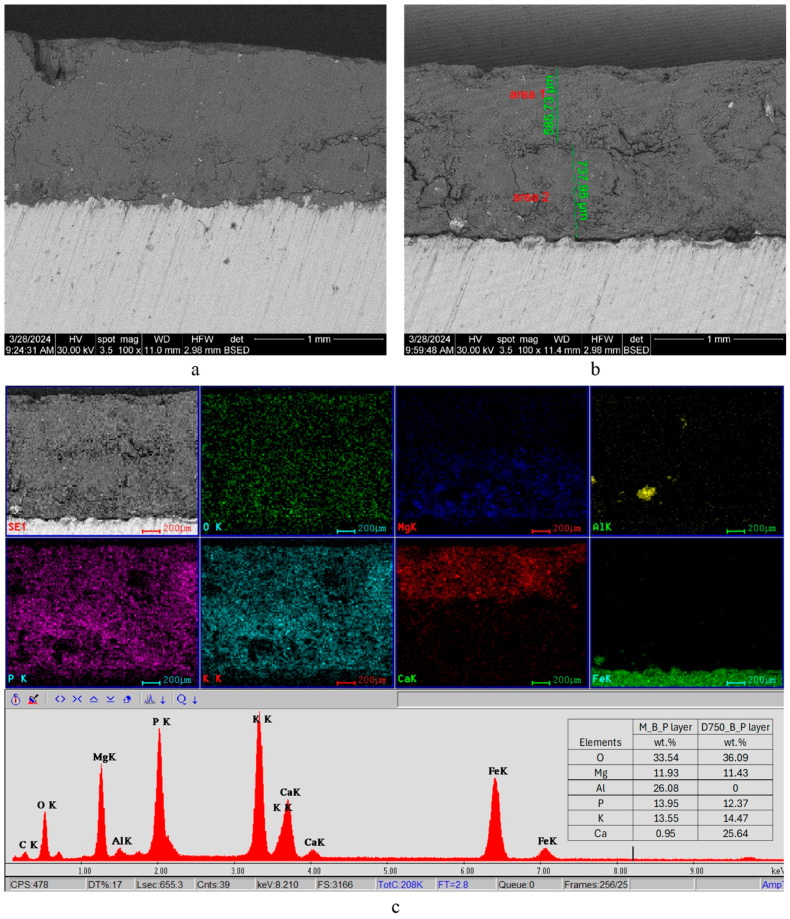
SEM images (**a**,**b**) and EDX analysis (**c**) of the metallic plate coated first with a layer of M_B_P cement, followed by a layer of DF750_P cement. Insert in (**c**) shows the elemental composition assessed by EDX on micro areas of the M_B_P layer and DF750_B_P layer.

**Figure 9:**
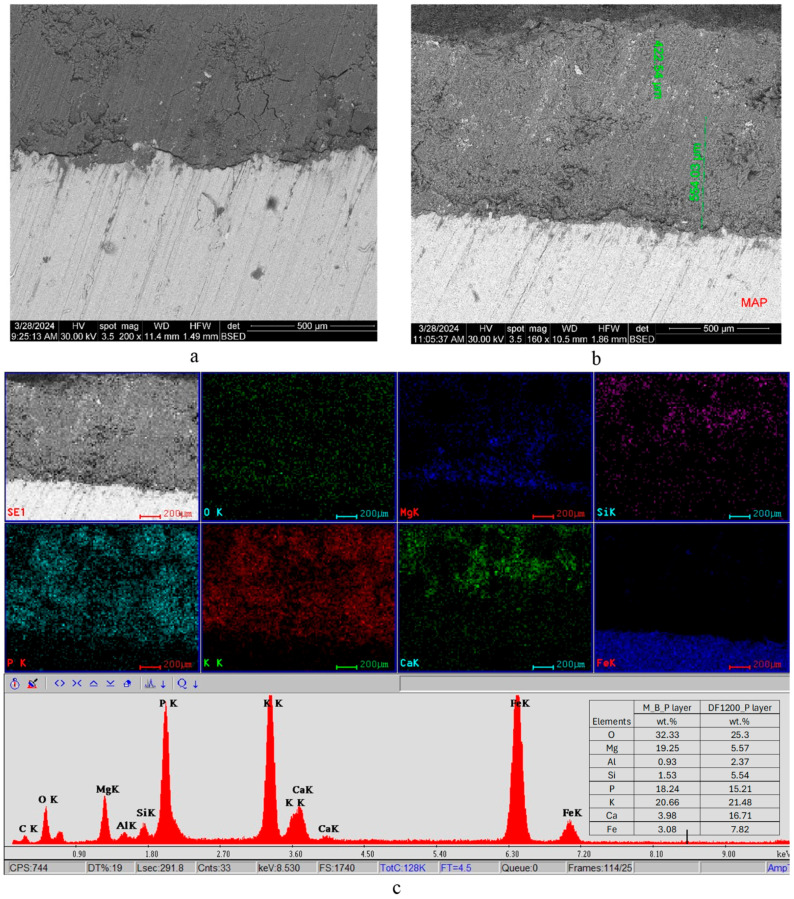
SEM images (**a**,**b**) and EDX analysis (**c**) of the metallic plate coated first with a layer of M_B_P cement, followed by a layer of DF1200_P cement. Insert in (**c**) shows the elemental composition assessed by EDX on micro areas of the M_B_P layer and DF1200_B_P layer.

The SEM and EDX analyses conducted on these specimens (Figure 8 and Figure 9) bring information referring to the elemental composition of the substrates, their thickness, and the compatibility between the M_B_P substrate and the metallic plate, respectively, its compatibility with DF1200_P and D750_B_P layers.

The first layer placed on the steel plate, i.e., the magnesium phosphate cement (M_B_P), has a thickness comprised between 550 and 910 μm. The second layer deposited on the M_B_P one consists of D750_B_P (Figure 8) and, respectively, DF1200_P (Figure 9).

A continuous and dense interfacial transition zone, indicative of good compatibility, can be observed between the M_B_P coating and the surface of the steel plate (Figure 8a). Additionally, a dense and continuous interface is visible between the M_B_P and D750_B_P layers (Figure 8b).

From the elemental maps (Figure 8c), the strong presence of Fe in the metallic plate can be observed, as well as the presence of Ca mainly in the superior layer (based on calcined dolomite); magnesium, potassium, and phosphorus elements are present in both layers, due to the formation of the KMgPO_4_·6H_2_O in both phosphate cements.

In the case of the steel plate coated first with a layer of M_B_P and with a superior layer of DF1200_P, one can also assess a continuous interfacial transition zone between these two layers, i.e., the studied cements have good compatibility (Figure 9a,b). The elemental distribution in this specimen is almost similar with the one previously assessed for D750_B_P cement, with the difference that in the DF1200_P layer, one can also assess Si; the provenience of this element is due to the presence of fly ash in the mixture used to obtain the precursor DF1200.

Table 4 shows the visual aspect of the coatings applied on the steel plates before, during, and after direct contact with the flame.

In the case of the M_B_P cement, the reduced water dosage (water to solid ratio of 0.2) allowed us to obtain an even and consistent coating devoid of phase separations or large cracks, which stayed on the metal substrate before, during, and after the contact with the flame.

After being subjected to direct flame, on the superior layer of the M_B_P + DF1200_P coating, one can observe visible cracks; nevertheless, the layer remains attached to the steel plate.

For the M_B_P + D750_B_P specimen, discoloration was observed in the area exposed to the flame, along with cracking attributed to material contraction. However, the coating remained well adhered to the steel plate surface.

Although these results are encouraging, the cracking observed in several coatings after 30 or 60 min of flame exposure may compromise their long-term performance. Therefore, future studies should consider implementing standardized testing protocols to assess fire performance.

To enhance the performance of these cements under direct flame exposure, expanded perlite was incorporated into the phosphate cement formulation in the case of the D750_3EP_B_P specimen; this porous material can contribute to further reducing the temperature of the metallic substrate, mainly due to its low thermal conductivity. When EP was incorporated in the D750_B_P cement paste, the bulk density decreased with 3 ± 0.2%.

This material was coated directly on the steel plate (D750_3EP_B_P specimen) or on an intermediate magnesium phosphate coating (M_B_P + D750_3EP_B_P specimen). In both cases, the thickness of the coating was greater (10–11 mm) when compared to the first three types of specimens (without EP) previously presented (medium thickness being 2 mm).

**Figure 10:**
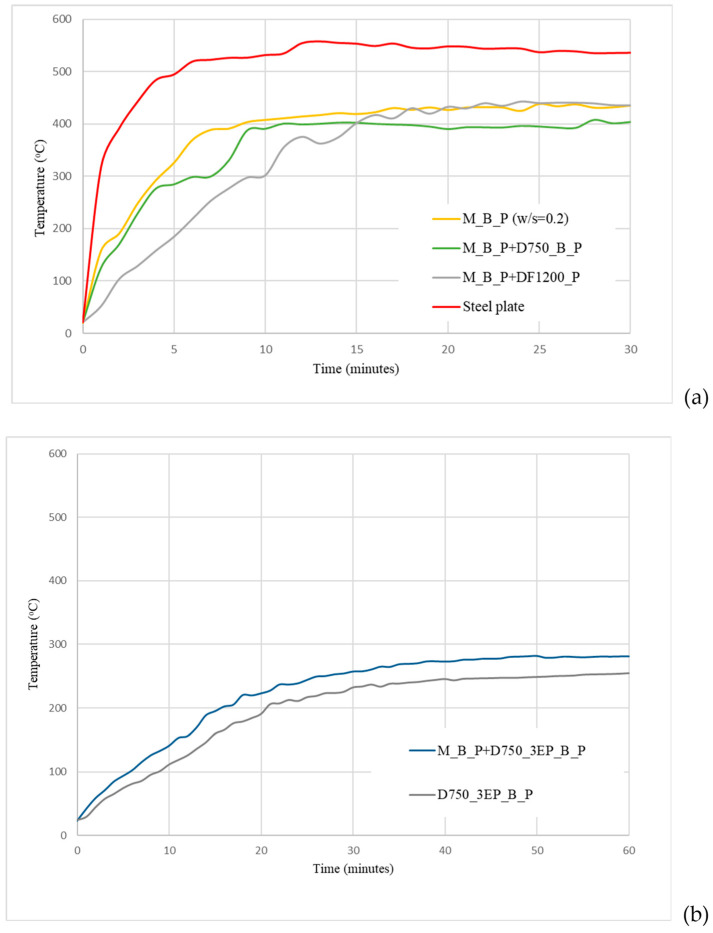
Steel substrate temperature vs. time for the steel plates with/without different coatings during fire test: (**a**) coatings without expanded perlite and (**b**) with expanded perlite.

During the fire tests, the temperature of the steel substrate was monitored, and the corresponding results are presented in Figure 10.

As illustrated in Figure 10a, the application of phosphate cement coatings on steel plates reduces both the rate of temperature increase during the first 10 min of direct flame exposure and the maximum temperature reached.

The increase in the coating thickness (in the case of D750_3EP_B_P and M_B_P + D750_3EP_B_P specimens) has a significant role in decreasing the rate of the temperature increase during the first 10–15 min of the fire test (Figure 10b). Moreover, due to the presence of porous expanded perlite, with low thermal conductivity, the thermal insulation properties of these coatings are increased.

The lower rate of the temperature increase for the coating based on D750_3EP_B_P as compared with the one with a supplementary M_B_P layer (i.e., M_B_P + D750_3EP_B_P) could be due to the lower heat absorption associated with the endothermic effects of the phosphate cement based on M as compared with the one based on D750 (which also contains CaCO_3_) [9]. The presence of this supplementary layer (M_B_P) in the M_B_P + D750_3EP_B_P coating could contribute to the decrease in the heat consumption in the endothermic processes, which occur during the heating of this coating.

Additionally, it can be assumed that the porous structure of expanded perlite (see Figure 2) could contribute to reduced shrinkage, thereby enhancing adhesion to the steel surface and preventing delamination during and after fire testing (see Table 4).

## 4. Conclusions

Phosphate cements based on dolomite thermally treated at various temperatures were developed in this study. Replacing dead burned magnesia with calcined dolomite in the formulation of phosphate cements can help reduce CO_2_ emissions, as dolomite requires significantly lower calcination temperatures compared to dead burned magnesia.

Among the tested formulations, the phosphate cement based on dolomite thermally treated at 750 °C exhibited the highest compressive strength.

When exposed to direct flame, the phosphate coating protected the steel by limiting the temperature increase and reducing the plateau temperature compared to the uncoated plate. All phosphate cement coatings remained attached to the steel plate surface during and after direct contact with the flame up to 60 min.

The inclusion of expanded perlite in the CMPCs based on dolomite calcined at 750 °C, combined with the increased coating thickness, significantly reduced the rate of the temperature rise in the steel substrate. Additionally, the porous structure of expanded perlite may help reduce shrinkage and prevent coating delamination during and after fire testing.

While the results obtained are encouraging, future research should be performed by incorporating standardized fire testing procedures, evaluating long-term performance, and conducting a comprehensive analysis of the economic viability of the proposed materials.

## Figures and Tables

**Table 1:** The composition (%wt.) of the studied phosphate cements.

Specimen	MgO (%)	D750 (%)	DF1200 (%)	Expanded Perlite (EP) (%)	KH_2_PO_4_ (%)	Borax (%)	Water to Solid Ratio ^1^
M_B_P	36	0	0	-	61	3	0.3 or 0.2
D750_B_P	-	58	0	-	39	3	0.3
DF1200_P	-	-	60	-	40	-	0.15
DF1200_B_P	-	-	58	-	39	3	0.15
D750_3EP_B_P	-	55	-	3	39	3	0.3

^1^ Considering also the water from borax decahydrate.

**Table 2:** The initial setting time (IST) and the final setting time (FST) of the studied phosphate cements.

Setting Time (min)	M_B_P	D750_P	D750_B_P	DF1200_P	DF1200_B_P
IST	6	4	53	70	>205
FST	9	7	62	149	Nd ^1^

^1^ Nd—not determined.

**Table 3:** The visual aspect of the specimens before and after the thermal treatment in the furnace.

Cement	The Specimens Before and After the Thermal Treatment	Comments
M_B_P (water to solid = 0.3)	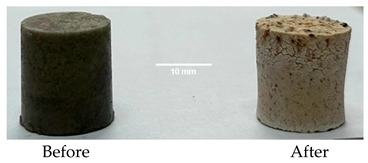	Expansion and cracking visible on the surface layer of the specimen; color change.
D750_B_P	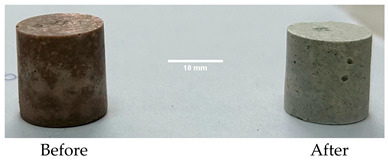	Shrinkage of the specimen; color change.
DF1200_P	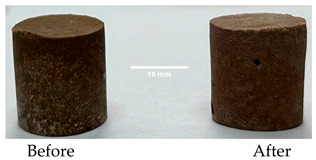	No significant changes were visibly observed.

**Table 4:** Macroscopic characteristics of the coated steel plates.

Binder Mixture	Before Test	During Test	After Fire Test
M_B_P (water to solid ratio = 0.2)	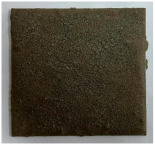	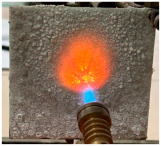	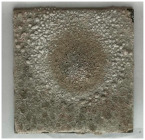
M_B_P + DF1200_P	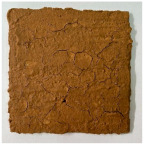	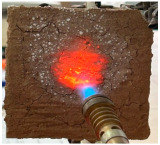	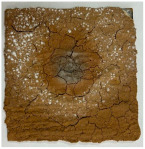
M_B_P + D750_B_P	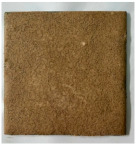	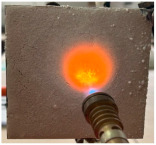	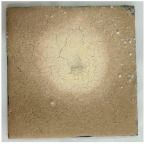
M_B_P + D750_3EP_B_P	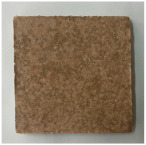	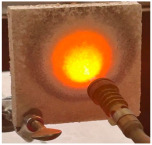	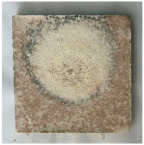
D750_3EP_B_P	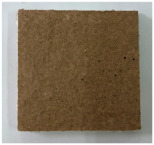	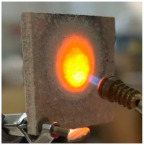	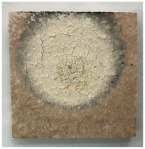

## Data Availability

The original contributions presented in this study are included in the article. Further inquiries can be directed to the corresponding author.
